# Complete Genome Sequence of Acinetobacter pittii BHS4, Isolated from Air-Conditioning Condensate in Hong Kong

**DOI:** 10.1128/MRA.00880-21

**Published:** 2021-10-21

**Authors:** B. H. Saunders, G. K. K. Lai, S. D. J. Griffin, F. C. C. Leung

**Affiliations:** a Shuyuan Molecular Biology Laboratory, The Independent Schools Foundation Academy, Hong Kong, Hong Kong SAR, China; University of Arizona

## Abstract

Acinetobacter pittii is widespread in the environment, and the Acinetobacter calcoaceticus*-baumannii* complex, to which it belongs, is a major cause of hospital-acquired pneumonia and bacteremia. *A. pitti* BHS4 was isolated from an air-conditioning unit in Hong Kong and its complete genome sequence (3,901,980 bp; GC content, 38.79%) established through hybrid assembly.

## ANNOUNCEMENT

T he Acinetobacter calcoaceticus-*baumannii* complex is a major cause of hospital-acquired pneumonia and bacteremia, with A. baumannii designated priority 1 on the WHO Priority Pathogens List (PPL) (1, 2). Acinetobacter species are known to accommodate a range of environmental conditions, and multidrug-resistant strains are increasingly being encountered (3–7). Recently, outbreaks of carbapenem-resistant Acinetobacter infections have been reported in COVID-19 intensive care unit (ICUs) (8, 9), with contaminated aerosols and ventilation units the likely sources (10, 11). Acinetobacter pittii is a Gram-negative, aerobic, nonmotile coccobacillus widely distributed in water, soil, and occasionally food stocks (12–16). While A. baumannii is the most common species in most regions, A. pittii and A. nosocomialis appear more prevalent in Southeast Asia (17).

BHS4 was isolated from water dripping from an air-conditioning unit in Mong Kok, Hong Kong. A 100-μl aliquot of the water sample was initially spread onto LB agar and incubated overnight at 37°C. Selected cream-colored colonies were passaged 10 times on LB agar and finally overnight in LB broth before genomic DNA (gDNA) extraction using Invitrogen’s PureLink genomic DNA minikit. Paired-end short-read sequencing libraries were prepared using the Nextera XT DNA library preparation kit and sequenced using the Illumina MiSeq platform with v3 chemistry (2 × 300 bp). Adapter sequences were removed using Trimmomatic v0.32 (18) and the reads quality filtered and trimmed. The resulting Illumina data set contained 1,204,982 read pairs with an average length of 297 bp (approximately 358 Mbp). Long-read libraries, prepared from the same extracted DNA using a gDNA rapid barcoding kit (SQK-RBK004), were sequenced using a SpotON flow cell vR9 and MinION sequencer, with data acquisition using MinKNOW v3.1.8 software and base calling using Guppy v2.1.3 (all from Oxford Nanopore). The final long-read data set, trimmed using Porechop v0.2.4 (19, 20), totaled 488,644 reads (3.07 Gbp) with a median length of 15,833 bp (*N*_50_, 60,789 bp). Default parameters were used for all software unless otherwise specified.

Assembly of the short reads using Newbler v2.7 (Roche Diagnostics) suggested a draft genome of ∼3.85 Mbp, based on 217 contigs at an average depth of 96×. However, a complete genome sequence was generated by combining the Illumina and MinION data sets using Unicycler v0.4.3 (21), yielding a circular chromosome of 3,894,835 bp and a circular plasmid of 7,145 bp, which were submitted to the NCBI PGAP v5.0 for annotation (22).

Multilocus sequence typing (MLST) of the BHS4 chromosome found proximity to Acinetobacter pittii IEC338SC (GenBank accession number CP015145.1), with average nucleotide identity of 96.9% (23, 24). In the plasmid, the nucleotide identity of the *repB* gene corresponds with Acinetobacter baumannii plasmid homology group GR3 (25) and with the recently defined plasmid lineage LN-16 (26). It encodes the *higBA2* toxin-antitoxin operon, shown to enable a response to environmental stress (27). In antimicrobial susceptibility tests, BHS4 demonstrated resistance to vancomycin and cephalothin (30-μg disks; Liofilchem) but not to ampicillin, Augmentin, cefepime, or ertapenem, despite the presence of the beta-lactamases OXA-421 and ADC-20 (28).

### Data availability.

The complete genome sequences and raw sequence data for A. pittii BHS4 are available through NCBI under BioProject accession number PRJNA729870, GenBank accession numbers CP075323 (chromosome) and CP075324 (plasmid), and SRA accession numbers SRX11971290 (Illumina MiSeq) and SRX11971291 (MinION).
